# Enriched Environment at Work and the Incidence of Dementia: Results of the Leipzig Longitudinal Study of the Aged (LEILA 75+)

**DOI:** 10.1371/journal.pone.0070906

**Published:** 2013-07-26

**Authors:** Francisca S. Then, Melanie Luppa, Matthias L. Schroeter, Hans-Helmut König, Matthias C. Angermeyer, Steffi G. Riedel-Heller

**Affiliations:** 1 Institute of Social Medicine, Occupational Health and Public Health, University of Leipzig, Leipzig, Germany; 2 LIFE – Leipzig Research Center for Civilization Diseases, University of Leipzig, Leipzig, Germany; 3 Max-Planck-Institute for Human Cognitive and Brain Sciences & Consortium for Frontotemporal Lobar Degeneration, Leipzig, Germany; 4 Department of Medical Sociology and Health Economics, University Medical Centre Hamburg-Eppendorf, Hamburg, Germany; 5 Center for Public Mental Health, Gösing am Wagram, Austria; 6 Department of Public Health, University of Cagliary, Cagliary, Italy; Federal University of Rio de Janeiro, Brazil

## Abstract

**Background:**

The high incidence of cognitive impairments in the aging population together with the challenges it imposes on health systems raise the question of what effect working life has on cognitive abilities. Animal models have demonstrated that so called enriched environments protect against neurodegenerative diseases, such as dementia. The aim was to investigate the impact of enriched environment at work on the incidence of dementia.

**Methods:**

The Leipzig Longitudinal Study of the Aged (LEILA 75+) is an ongoing representative population cohort study that examines cognitive functioning and dementia in individuals aged 75 years and older. The participants’ occupational information was matched to O*NET SOC codes and the relevant job descriptors were used to create occupational context indices describing enriched environment at work.

**Results:**

Results of logistic regression modeling suggest that a higher level of the index *Executive* was associated with a lower risk of incident dementia (odds ratio  = 0.61, 95% confidence interval  = 0.47–0.79, p<0.001). Adjustment for various confounders did not alter the association. The cognitive stimulation indices were only significant in univariate analysis. The *Novelty*-index remained non-significant.

**Conclusions:**

The results suggest that occupational contexts enriched with independent planning/performance of work tasks might decrease the risk of developing dementia. A protective effect of enriched environment at work in general, namely high cognitive stimulation or confrontation with new tasks, could not be confirmed by the results.

## Introduction

Prevalence of dementia affects more than 24 million people worldwide with an estimated increase of 4.6 million new cases every year [1]. The annual costs of care per patient average out at US$9,829 in the U.S.A. [2] and €7,820 in Europe [3]. Whereby annual costs more than double for severe dementia stages [4]. Due to the high impact that dementia has on the society, it is essential to come to a thorough understanding of its pathogenical processes in order to establish prevention methods that decrease the risk to develop dementia or delay the onset of the disease.

With respect to dementia, a great number of risks and protective factors have been identified [5]. However, most of the information on these factors has been collected in old age. It is very likely that at that time pathogenical processes have already started. Therefore it is necessary to look at factors in midlife. Recent publications with primary evidence suggest that in particular characteristics of the occupational context affect the incidence of dementia: Mental demands in the work place significantly enhance cognitive functioning in old age [6] and protect against dementia [7]. Specifically, a high level of complexity when working with data, like analyzing, is decreasing the risk of cognitive impairment [8]–[10]. Also, an elevated level of concentration and precision prevents from cognitive deterioration [11]. Since the occupational context substantially determines the quantity and quality of intellectual activities, it is to be expected that it has consequences on cognitive deterioration in old age. The theory of cognitive reserve assumes that achieving a high cognitive ability level in midlife can protect from the effects of neurodegenerative disease, like Alzheimer’s disease [12]. In this way, an occupational context that provides the “right” intellectual demands contributes fundamentally to a greater cognitive reserve. However, the exact process by which that happens still remains unclear.

Insights in the neurochemical processes, which are involved in particular environmental stimulation patterns, are gained through animal studies. Animal models of brain disorders have demonstrated that so called ‘enriched environments’ protect against neurodegenerative diseases, such as dementia, by up-regulating synaptogenesis and synaptic plasticity as well as by epigenetic mechanisms [13]. Findings in human studies, as far as they have been conducted, support the idea that these neurochemical processes also play an important role in the development of dementia [14]. For instance, high BDNF (brain-derived neurotropic factor) serum levels have been associated with slower cognitive decline [15]. In order to understand the association between the environmental context that the individual is exposed to and the processes that lead to cognitive decline and dementia, we need to close the gap between animal studies and human research. To the best of our knowledge, no other study has so far attempted to make this connection by taking a translational stance. For that purpose, the current study imitates the characteristics of an enriched environment, as it is found in animal studies, with corresponding characteristics in typical human environmental contexts. The use of characteristics of the occupational context was chosen, because a) for that context relatively objective measurements are available, b) work conditions are comparable, c) the variance in occupational contexts is high enough, and d) the point of time (when being active in workforce) lays evidently ahead of the expected point of onset of dementia.

The study investigated the association between enriched environment as found in the workplace and the incidence of dementia in a longitudinal cohort design.

## Methods

### Ethics Statement

The Ethics committee of the Leipzig University (Ethik-Kommission an der Medizinischen Fakultät der Universität Leipzig) approved the study. Written informed consent of the participants was obtained before assessment. The participants’ capacity to consent was assessed by trained and experienced physicians and psychologists before starting the interview. If a person was found incapable to consent, informed consent was obtained from the next of kin, care taker, or guardian on the behalf of the participant.

### Study Design

**Figure 1:**
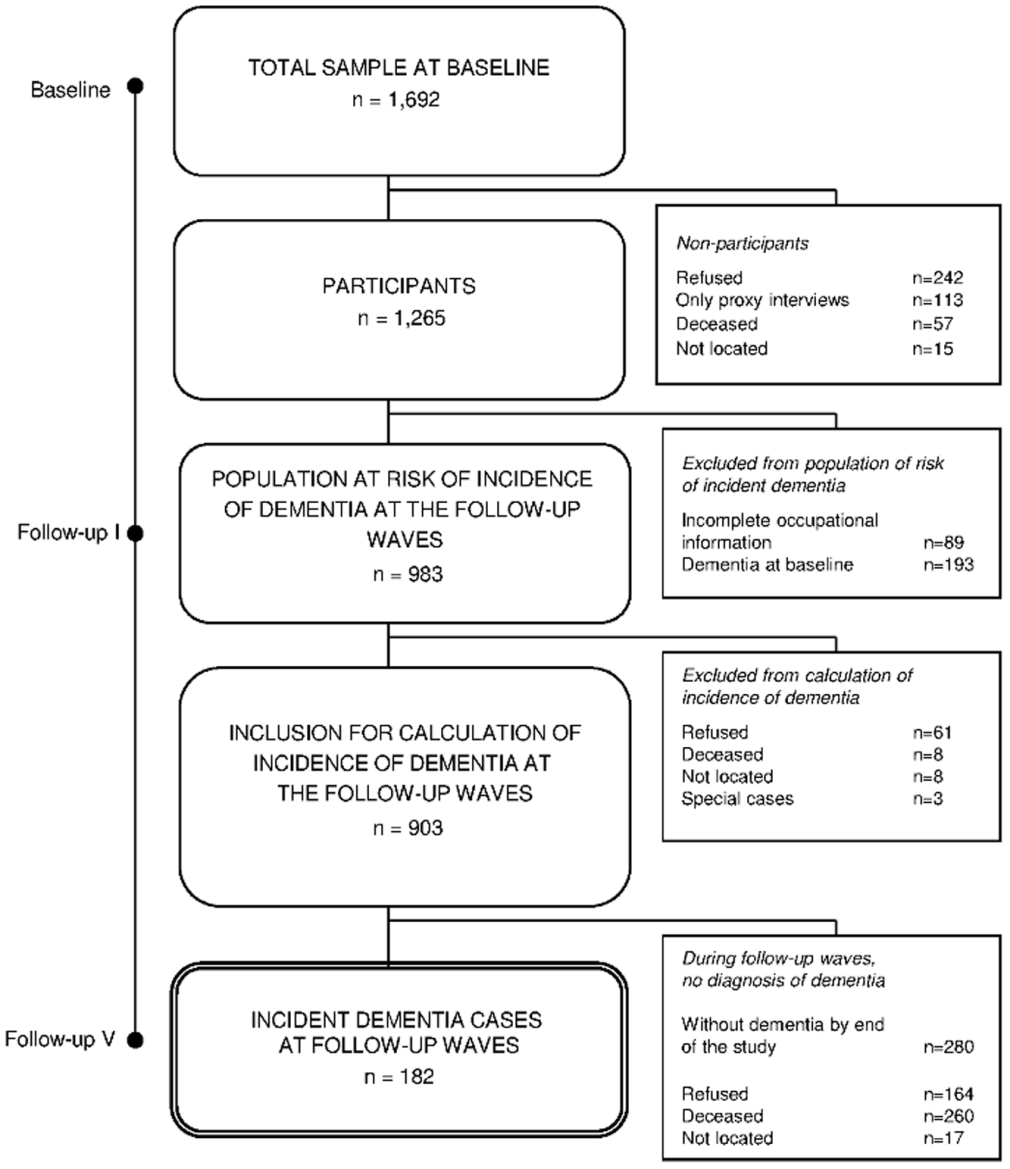
Sampling Frame of the Participants.

Participants were enrolled in the Leipzig Longitudinal Study of the Aged (LEILA 75+), a population-based study on the epidemiology of dementia. The study design is described elsewhere in detail [16]. 1,692 individuals aged 75 years and over in the city area Leipzig, Germany, were included at baseline (1997–1998). 242 (14.2%) of the participants refused to participate, 57 (3.4%) died, and 15 (0.9%) could not be located. A total of 1,265 (74.6%) individuals completed the neuropsychological assessment. Study participants did not differ significantly from nonparticipants with regard to gender, age, or marital status [16]. Individuals were followed for eight years and examined six times in this period (five follow-ups at an 18-month interval). 89 of the participants did not provide sufficient occupational information, and 193 participants were diagnosed with dementia according to DSM-IV criteria at baseline. To be able to identify an effect on the incidence of dementia, prevalent cases were excluded from the initial analysis. For 80 individuals (8%), there was no follow-up information available. The final sample consisted of 903 participants (see Figure 1 for flow of participants).

### Assessment of Dementia

Structured clinical interviews were conducted in the participants’ home environment by trained psychologists and physicians. The SIDAM (structured interview for the diagnosis of dementia of the Alzheimer type, multi-infarct dementia and dementias of other etiology according to ICD-10 and DSM-III-R), a neuropsychological test battery that covers six areas of cognitive functioning and includes the 30 items of the Mini-Mental State Examination (MMSE) [17] was administered. Additionally, information on cognitive and psychosocial functioning was obtained through fully structured proxy interviews. Diagnosis of dementia was made in consensus conferences of physicians and psychologists for each participant based on the DSM-IV criteria.

### Occupational Information

The participants’ occupational information was collected during the personal interview. The longest held occupation was translated to English and coded according to the O*NET standard occupational classification (SOC, http://www.onetonline.org/). The O*NET database is the primary source of occupational information provided by the US Department of Labor/Employment and Training Administration (USDOL/ETA). It contains hierarchical taxonomic occupation-specific descriptors and is continually updated. Coding of the participants’ occupations by a second blinded rater indicated a high interrater reliability (Cohen’s kappa  = 0.79). Non-consensus was resolved by matching of the occupational criteria entry level requirements, daily tasks, level of responsibility at work, and the amount of machinery used to relevant O*NET SOC Code. When participants reported to have held two or more occupations for long periods of their lives, averages of the occupation-specific descriptors were calculated.

### Operationalization Enriched Environment at Work

The conceptualization of enriched environment was based on a literature search in PubMed and PsycInfo for journal articles using the term “enriched environment” or “environmental enrichment” in title or abstract. Characteristics that defined certain environments as “enriched” were extracted from relevant articles. Findings indicate that in an enriched environment, the animal is commonly exposed to new objects of different characteristics (shape, texture, colors) that are rearranged and changed at frequent intervals (e.g. [18]–[20] ). The exploration of these objects through the animal is completely voluntary and self-paced. Essentially, the objects in the enriched environment are thought to provide enhanced sensory and cognitive stimulation compared to ordinary conditions [21]. The observed characteristics were used to create three categories that describe an enriched environment: (a) the confrontation with new stimuli, (b) a high number of objects that stimulate the use of higher cognitive skills, and (c) the opportunity for the individual to carry out tasks independently. It is expected that these categories provide an adequate degree of comparability between animal and human environments. Transferring the categories to the human occupational context, four indices of enriched environment at work were created:

–*Novelty*, indicating the intensity of confrontation with new stimuli
–*Fluid*, indicating the cognitive stimulation of work tasks on fluid intelligence
–*Verbal*, indicating the cognitive stimulation of work tasks on crystallized intelligence
–*Executive*, indicating the level of independent planning and performance of work tasks

Descriptors of work activities and work context in the O*NET 16.0 database were screened for concept validity of the four indices. Only O*NET variables with a standard error <0.5 were considered for inclusion in further analysis. Eligible descriptors were included in an index only, if the two-tailed correlation with all other descriptors in the index was significant (p<.01). Descriptors with negative correlation were reversed. Cronbach’s alpha coefficients of the indices created were 0.79 for *Novelty*, 0.89 for *Fluid,* 0.97 for *Verbal*, and 0.96 for *Executive*, respectively. The detailed content of the descriptors of the indices can be found in Appendix S1. A higher score in the index indicates a higher level of demands in this occupational context. In this sense, an increase of one ‘index unit’ means an average overall increase of the level of demands –for example, from a moderate level to a high level – of the occupational context captured by the particular index.

### Statistical Analyses

The characteristics of individuals who remained free of dementia and those who developed dementia were compared using Mann-Whitney-U-Test, for continuous variables, and Pearson’s chi-square, for categorical data. The association between the occupational context indices and dementia was assessed using binary logistic regression models. Logistic regression estimates the probability of events via odds ratios, and can be used even when some of the variables are not normally distributed [22], [23].

Models included the occupational context index, age (continuous), and education (low/middle/high according to [24] ). Model 2 included additionally gender (male/female), marital status (married/single/divorced/widowed), history of depression (yes/no), and living situation (alone/with somebody/nursing home). Covariate information came from the baseline assessment.

To get clear incident rates, prevalent dementia cases were excluded from principal analysis. Moreover, since the independent variables refer to the time of being an active part of the workforce, we can proceed from the assumption that the participant did not have dementia nor were seriously cognitive impaired at that time.

All statistical analyses were performed using SPSS 20 software.

## Results

The participants without dementia were compared to those who developed dementia during the study period. A total of 182 individuals were diagnosed with dementia. Demented participants were significantly older, more often women, more frequently widowed, more likely to be living in a nursing home, and had a greater likelihood to have had a stroke (Table 1). The level of *Executive*, *Verbal*, and *Fluid* work task demands was significantly lower in individuals that developed dementia.

**Table 1:** Demographics and occupational context indices for dementia-free participants and dementia cases.

Characteristic		Non-demented participants		Dementia cases		P-value
		N	%	N	%	
Gender	male	201	27.8%	26	14.4%	0.000*
	female	522	72.2%	154	85.6%	
Education	low	463	64.2%	128	71.1%	0.109
	middle	165	22.9%	38	21.1%	
	high	93	12.9%	14	7.8%	
Marital status	married	220	30.4%	25	13.9%	0.000*
	single	65	9.0%	13	7.2%	
	divorced	65	9.0%	17	9.4%	
	widowed	373	51.6%	127	69.4%	
Living situation	with somebody	267	36.9%	39	21.7%	0.000*
	alone	409	56.6%	102	56.7%	
	nursing home	47	6.5%	39	21.7%	
Stroke	no	685	94.7%	162	90.0%	0.018*
	yes	38	5.3%	18	10.0%	
Heart attack	no	655	90.6%	168	94.4%	0.107
	yes	68	9.4%	10	5.6%	
Diabetes	no	563	77.9%	132	73.3%	0.196
	yes	160	22.1%	48	26.7%	
Depression	no	653	90.6%	165	92.2%	0.503
	yes	68	9.4%	14	7.8%	
Alcohol	no	81	11.3%	30	16.8%	0.097
	normal	594	82.8%	142	79.3%	
	at risk	42	5.9%	7	3.9%	
Smoking	no	469	65.3%	137	76.5%	0.016*
	former	194	27.0%	32	17.9%	
	current	55	7.7%	10	5.6%	
		mean	st.d.	mean	st.d.	P-value
Age		80.88	4.63	83.99	4.76	0.000*
SIDAM score		47.18	5.14	42.81	5.58	0.000*
*Novelty*-Index		2.98	0.70	2.89	0.76	0.048
*Executive*-Index		2.78	0.73	2.55	0.61	0.001*
*Verbal*-Index		3.15	0.84	2.94	0.77	0.000*
*Fluid*-Index		2.61	0.45	2.51	0.44	0.005*

In univariate logistic regression modeling, the occupational context indices *Executive* (OR 0.61, CI 95% 0.47–0.79), *Verbal* (OR 0.74, CI 95% 0.61–0.90) and *Fluid* (OR 0.59, CI 95% 0.41–0.87) were significantly associated with the incidence of dementia. The index *Novelty* was non-significant (Table 2). The significance of the indices *Verbal* and *Fluid* disappeared when adjusted for age and education (model 1); the models themselves however remained significant predictors of demented cases. The index *Executive* stayed significant for model 1 (OR 0.68, CI 95% 0.51–0.91) and model 2 (OR 0.73, CI 95% 0.53–0.99). Each model that included the index *Executive* was significant in predicting dementia at a p<0.001-level. Adding any other covariate did not alter the nature of the association. According to the results, higher values of the occupational context index *Executive* significantly protected against the incidence of dementia.

**Table 2:** Logistic Regression Models of the association between the occupational context indices and the incidence of dementia in individuals 75 years and older.

	Univariate		Model 1^§^		Model 2#	
	OR (95% CI)	P-value	OR (95% CI)	P-Value	OR (95% CI)	P-Value
*Novelty*-Index	0.84 (0.66–1.07)	0.152	0.95 (0.73–1.23)	0.694*	1.15 (0.87–1.52)	0.315*
*Executive*-Index	**0.61 (0.47–0.79)**	**0.000** *	**0.68 (0.51–0.91)**	**0.008** *	**0.73 (0.53–0.99)**	**0.044** *
*Verbal*-Index	**0.74 (0.61–0.90)**	**0.003** *	0.87 (0.70–1.08)	0.194*	0.91 (0.72–1.15)	0.415*
*Fluid*-Index	**0.59 (0.41–0.87)**	**0.008** *	0.73 (0.49–1.07)	0.107*	0.99 (0.63–1.57)	0.972*

*Notes* ^§^model includes the predictor, age, and level of education;

# model includes the predictor, age, level of education, gender, marital status, history of depression, and living situation;

* complete model is significant at a p<0.01 level; OR, odds ratio/effect coefficient Exp(B); CI, confidence interval.

Factors of model 2 that independently added to the predictive strength were age, gender, and living situation (p<0.05). Thus, being older, being a woman, and living in a nursing home increased the risk of developing dementia.

Including prevalent dementia cases in the analyses raised the significance of the index *Executive* for model 1 (OR 0.62, CI 95% 0.48–0.80, p<0.001) and model 2 (OR 0.66, CI 95% 0.50–0.88, p = 0.004). Moreover, the index *Verbal* remained significant in model 1 (OR 0.62, CI 95% 0.49–0.80, p<0.001) and model 2 (OR 0.64, CI 95% 0.49–0.83, p = 0.001). Nonetheless, it did not change the results for the indices *Novelty* and *Fluid*.

Excluding housewives from the analysis did not modify the associations.

## Discussion

The results show that high levels of the occupational context index *Executive*, specifying the possibility to independently plan and carry out work tasks, actually decreased the risk of dementia. Analyses indicate that the observed effect is highly consistent and contributes independently to the predictive power of the models. Including prevalent cases in the analysis intensifies the statistical significance. Looking at the odds ratios, this might be due to an increase in power. The current study also revealed a non-significant relationship between the occupational context indices *Novelty, Fluid, Verbal*, and the risk of developing dementia. Even though the indices for cognitive stimulation in the work place, *Verbal* and *Fluid*, were statistical significant in univariate analysis, they lost significance when adjusted for confounders. The index *Verbal* remained only a significant protector against dementia, when prevalent cases were included in the analysis. This suggests that the level of stimulation of the verbal intelligence by the occupational context has only an impact on the incidence of dementia before the age of 75, because all incident cases have been at least 75 years old by the beginning of the study. The current study examined only the risk of getting dementia. The extent to which intellectual stimulation within the occupational context is actually capable of delaying the onset of dementia needs to be evaluated in further analyses, for example, via cox proportional hazards models or by calculating the person years at risk.

So far, published studies relating *Executive* demands (tasks with a high level of independent planning and performance) in the work place to dementia looked at job control. Analyses showed that high job control protects against dementia [25], [26]. It is likely that job control enhances autonomy, and the regular training of these particular abilities protects against cognitive decline in old age, as proposed by the use-it-or-lose-it hypothesis [27]. Moreover, the occupational context might help building up a cognitive reserve that later in life improves the ability to deal with the consequences of neurodegenerative pathologies. The results support the notion that having built up high functioning abilities of independent planning and performance of work tasks could operate like a cognitive reserve.

Moreover, the opportunity to be able to plan and perform work tasks independently (scoring high in the *Executive* index) has another benefit: In experimental studies, it has been shown that individuals who have control over their study time allocation outperformed others [28]. The authors observed that self-pacing, in the sense of being able to plan work tasks independently, led to an optimal work flow based on individual differences. In this way an individual can naturally prevent work overload and, hence, a stress reaction, which could contribute to a pathogenic process in the long-run.

The presented findings are an initial attempt of directly transferring a concept of animal studies to a human environment in a translational stance. In contrast to animal studies, where an enriched environment is considered neuroprotective, such a statement is not possible for the human work context based on the results. We observed distinguished effects with respect to the incidence of dementia: A high level of *Executive* demands potentially contribute to protect against dementia, but *Novelty,* namely the intensity of confrontation with new stimuli in the workplace, did not significantly affect dementia incidence. Animal studies on enriched environment implicitly assume that the more new objects are introduced, the better. The here demonstrated results suggest that it is not necessarily the nature of the presented stimuli that is protective, but the initiated behavior, namely the active exploration of the objects. Certainly, an aspect to consider is that humans and animals have dissimilar competencies. Competencies like inferential reasoning and abstract representations of underlying unobservable causal mechanisms are considered to be unique to humans [29]. It is unclear at this point of time how far competencies affect the process of transferring the concept of enriched environment to human contexts.

Despite the observed strength of the association, conclusions must be interpreted with great caution. The indices are based on classifications and not of the individual’s actual work environment. However, using classifications, such as the O*NET database, circumvents a possible recall bias in the elderly, partially demented, population. Unfortunately, the indices include only variables available in the O*NET database, and thus do not reflect the complete occupational exposure. Especially, since the O*NET database was developed for the North American market and further studies will have to validate the descriptors with respect to Europe. On the other hand, it is possible that the actual associations are stronger than observed in the current analyses, because the variance of the exposure variables is reduced due to the use of averaged database descriptors and the creation of indices. Longitudinal studies −20 to 50 years- that empirically assess detailed occupational exposure are necessary in order to validate the findings with respect to the incidence of dementia.

Further limitations include the loss of participants due to death and non-responders. A survival bias cannot be excluded, because there is no available evidence on whether the level of intellectual demands at work can influence mortality. Additionally, in order to exclude a possible reverse causality, future research should include inter-individual differences such as intelligence, capabilities, and personality.

Based on the here presented results, we come to two conclusions: First, having the goal in mind to diminish the prevalence rate of dementia in the long run, upcoming research should focus on environmental contexts enriched with opportunities to independent plan and perform tasks as modifiable protective factors against dementia. And second, further research should consider a differentiation between exploratory environments and novel environments. The here presented findings should be replicated and, if necessary, improved, preferably using sets of environmental characteristics that allow a comparison between animal and human settings. Moreover, comparisons between different intensity levels should be made so that a dose-response-relationship between environmental contexts and the risk of dementia can be established. In order to get a better understanding of underlying mechanisms such as spine morphogenesis and synaptic plasticity, it is essential to develop concordant animal and human models that allow for a high degree of comparison.

## Supporting Information

Appendix S1 O*NET descriptors included in the indices.
(DOCX)
