# Supplementary material for: Enriched Environment at Work and the Incidence of Dementia: Results of the Leipzig Longitudinal Study of the Aged (LEILA 75+)
Source: PLoS One. 2013 Jul 26;8(7):e70906. doi: 10.1371/journal.pone.0070906 (PMC3724805; doi:10.1371/journal.pone.0070906)
Supplement: Appendix S1 — O*NET descriptors included in the indices. (DOCX) [file pone.0070906.s001.docx]

Appendix S1

O*NET descriptors included in the indices:

*Index novelty*

The index for “novelty” consists of these O*NET descriptors:

1.B.1.f Conventional (reversed)

1.B.2.f Independence

1.C.4.c Adaptability/Flexibility

1.C.7.a Innovation

4.A.2.b.2 Thinking creatively (level)

4.A.2.b.2 Thinking creatively (importance)

4.C.3.b.7 Importance of Repeating Same Task (reversed)

*Index executive*

The index for “executive” consists of these O*NET descriptors:

4.A.2.b.4 Developing Objectives and Strategies (importance)

4.A.2.b.4 Developing Objectives and Strategies (level)

4.A.2.b.5 Scheduling Work and Activities (importance)

4.A.2.b.5 Scheduling Work and Activities (level)

4.A.4.a.7 Resolving Conflicts and Negotiating with Others (importance)

4.A.4.a.7 Resolving Conflicts and Negotiating with Others (level)

4.A.4.b.1 Coordinating the Work and Activities of Others (importance)

4.A.4.b.1 Coordinating the Work and Activities of Others (level)

4.A.4.b.4 Guiding, Directing, and Motivating Subordinates (importance)

4.A.4.b.4 Guiding, Directing, and Motivating Subordinates (level)

*Index verbal*

The index for “verbal” consists of these O*NET descriptors:

4.A.1.a.1 Getting Information (importance)

4.A.2.a.3 Evaluating Information (importance)

4.A.2.a.3 Evaluating Information (level)

4.A.2.b.3 Updating and Using Relevant Knowledge (importance)

4.A.2.b.3 Updating and Using Relevant Knowledge (level)

4.A.4.a.1 Interpreting the Meaning of Information (importance)

4.A.4.a.1 Interpreting the Meaning of Information (level)

4.A.4.b.6 Providing Consultation and Advice (importance)

4.A.4.b.6 Providing Consultation and Advice (level)

4.C.1.a.2.f Telephone

4.C.1.a.2.h Email

4.C.1.a.2.j Letters

*Index fluid*

The index for “fluid” consists of three clusters of O*NET descriptors:

Working memory: 1.A.1.d.1 Memorization (importance)

1.A.1.d.1 Memorization (level)

1.A.1.g.1 Selective Attention (importance)

1.A.1.g.1 Selective Attention (level)

1.A.1.g.2 Time Sharing (importance)

1.A.1.g.2 Time Sharing (level)

1.C.5.b Attention to Detail

4.A.1.a.2 Monitor Processes, Materials, Surroundings (level)

4.C.3.b.4 Importance of Being Exact or Accurate

Percept. Reasoning: 4.A.1.b.1 Identifying Objects, Actions, and Events (importance)

4.A.1.b.1 Identifying Objects, Actions, and Events (level)

4.A.1.b.3 Estimating the Quantifiable Characteristic (importance)

4.A.1.b.3 Estimating the Quantifiable Characteristic (level)

4.A.2.a.2 Processing Information (importance)

4.A.2.a.2 Processing Information (level)

4.A.2.a.4 Analyzing Data or Information (importance)

4.A.2.a.4 Analyzing Data or Information (level)

4.A.2.b.1 Making Decisions and Solving Problems (importance)

4.A.2.b.1 Making Decisions and Solving Problems (level)

Perceptual speed: 4.A.3.a.4 Operating Vehicles or Equipment (importance)

4.A.3.a.4 Operating Vehicles or Equipment (level)

4.C.3.d.3 Pace Determined by Speed of Equipment
